# Magnetofection approach for the transformation of okra using green iron nanoparticles

**DOI:** 10.1038/s41598-022-20569-x

**Published:** 2022-10-04

**Authors:** Naila Farooq, Laraib Ather, Muhammad Shafiq, Muhammad Shah Nawaz-ul-Rehman, Muhammad Haseeb, Tehmina Anjum, Qamar Abbas, Mujahid Hussain, Numan Ali, Syed Agha Armaghan Asad Abbas, Sehrish Mushtaq, Muhammad Saleem Haider, Saleha Sadiq, Muhammad Adnan Shahid

**Affiliations:** 1 Department of Biotechnology, Lahore Garrison University, P.O BOX. 54000, Lahore, Pakistan; 2 Department of Horticulture, Faculty of Agricultural Sciences, University of the Punjab, P.O BOX. 54590, Lahore, Pakistan; 3 Virology Lab, CABB University of Agriculture, Faisalabad, Pakistan; 4 Department of Plant Pathology, Faculty of Agricultural Sciences, University of the Punjab, P.O BOX. 54590, Lahore, Pakistan; 5 Department of Agronomy, Faculty of Agricultural Sciences, University of the Punjab, P.O BOX. 54590, Lahore, Pakistan; 6 Faculty of Agricultural Sciences, University of the Punjab, P.O BOX. 54590, Lahore, Pakistan; 7 Institute of Biochemistry, Biotechnology, and Bioinformatics (IBBB), The Islamia University of Bahawalpur, P.O BOX. 63100, Bahawalpur, Pakistan; 8 North Florida Research and Education Center, 155 Research Rd., Quincy, FL 32351 USA

**Keywords:** Biological techniques, Biotechnology, Cell biology, Genetics, Molecular biology, Plant sciences, DNA nanotechnology, Nanobiotechnology, Nanoscale materials, Techniques and instrumentation

## Abstract

Climate change, pesticide resistance, and the need for developing new plant varieties have galvanized biotechnologists to find new solutions in order to produce transgenic plants. Over the last decade scientists are working on green metallic nanoparticles to develop DNA delivery systems for plants. In the current study, green Iron nanoparticles were synthesized using leaf extract of *Camellia sinensis* (green tea) and Iron Chloride (FeCl_3_), the characterization and Confirmation was done using UV–VIS Spectroscopy, FTIR, SEM, and TEM. Using these nanoparticles, a novel method of gene transformation in okra plants was developed, with a combination of different Magnetofection factors. Maximum gene transformation efficiency was observed at the DNA to Iron-nanoparticles ratio of 1:20, by rotation of mixture (Plasmid DNA, Iron-nanoparticles, and seed embryo) at 800 rpm for 5 h. Using this approach, the transformation of the GFP (green fluorescent protein) gene was successfully carried out in *Abelmoschus esculentus* (Okra plant). The DNA transformation was confirmed by observing the expression of transgene GFP via Laser Scanning Confocal Microscope (LSCM) and PCR. This method is highly economical, adaptable, genotype independent, eco-friendly, and time-saving as well. We infer that this approach can be a potential solution to combat the yield and immunity challenges of plants against pathogens.

## Introduction

Advancement in medicine and the wide use of drugs has developed resistance in human and plant pathogens. Therefore, there is a need to develop new transgenic crop varieties that can sustain in the changing climate and can resist the pathogen attack. However, it requires a precise method that can successfully deliver foreign DNA into the plants. In this regard, over the nineteenth century, many methods of genetic transformation have been developed like the biolistic method (gene gun)^1^, electroporation^2^, sonoporation^3^, transfection^4^, magnetofection^5^, protoplast fusion^6^, microinjection^7^, vacuum infiltration^8^, and *Agrobacterium-*mediated transformation^9^, etc. Every genetic transformation method, whether the method is chemical, physical, or biological, has certain limitations^10^. For example, electroporation can damage the DNA or cause it to lose its integrity^11^. Similarly, *Agrobacterium*-mediated transformation is not a target-specific method^12^. All these limitations have urged biologists to purpose new solutions for DNA delivery.

All around the globe, there are high concerns regarding environment risk assessment of different genetically modified plants^13^. So, it’s important to move towards the techniques that pose minimal risk. Since last decade, Green Iron-nanoparticles (GINPs) are being synthesized and used in different fields such as medicine, antimicrobial activity, pollution control, and Azo-dye degradation, etc.^14–17^. Green synthesis is an alternative method for chemical and physical methods as it provides economic and environmental benefits^18^. However, its use in gene transformation for treating human diseases is quite new^19^. GINPs have the DNA binding capability that can be used for developing DNA delivery systems^20,21^. Indeed, this system uses the Magnetofection technique for gene transformation in the plants as well as in tumor cells^22^.

Recently, plant extracts are extensively used in the synthesis of GINPs that provide more stability to the metallic nanoparticles^23^. Valentine V. et al. in their study of the biosynthesis of iron oxide nanoparticles, suggested that the presence of organic acids (such as citric or oxalic acids) aids mainly in the stabilization of iron nanoparticles and plant having these organic acids can produce highly stable iron nanoparticles^24^. Many organic compounds (such as flavonoids, alkaloids, and saponins, etc.) are present in plant extracts that provide more solubility and compatibility to the GINPs^25^. Besides these, GINPs reduce the risk of environmental toxicity; as the coating of metallic particles with other non-biodegradable polymers can be harmful^26^. Hence, the metallic-nanoparticles prepared by using plant extract and used as DNA delivery system in plants is much more efficient as compared to the Magnetic Nanoparticles (MNPs) prepared with synthetic polymers.

In the current study, we have prepared the GINPs using leaf extract of *Camellia sinensis*. The optimization of Magnetofection was done to enhance DNA delivery via GINPs. We have successfully transformed the GFP gene in the *Abelmoschus esculentus* (Okra). Green tea leaves were used in the study because they contain organic acids (such as citric or oxalic acids), which aids in the production of stable iron nanoparticles. To revolutionize agriculture, this novel method can be used to transfer genes in plants.

## Materials and methods

### Plant materials

Green tea plant leaves were kingly provided by Arif Ahmed (Botanical Garden, University of the Punjab) and the Okra seeds were kindly provided by Dr. Atif Kamran (Seed Centre, Department of Botany, University of the Punjab). All the experimental work on plant material described in this study complies with the relevant institutional, national, and international guidelines and legislation.

### Synthesis and characterization of iron nanoparticles

**Figure 1:**
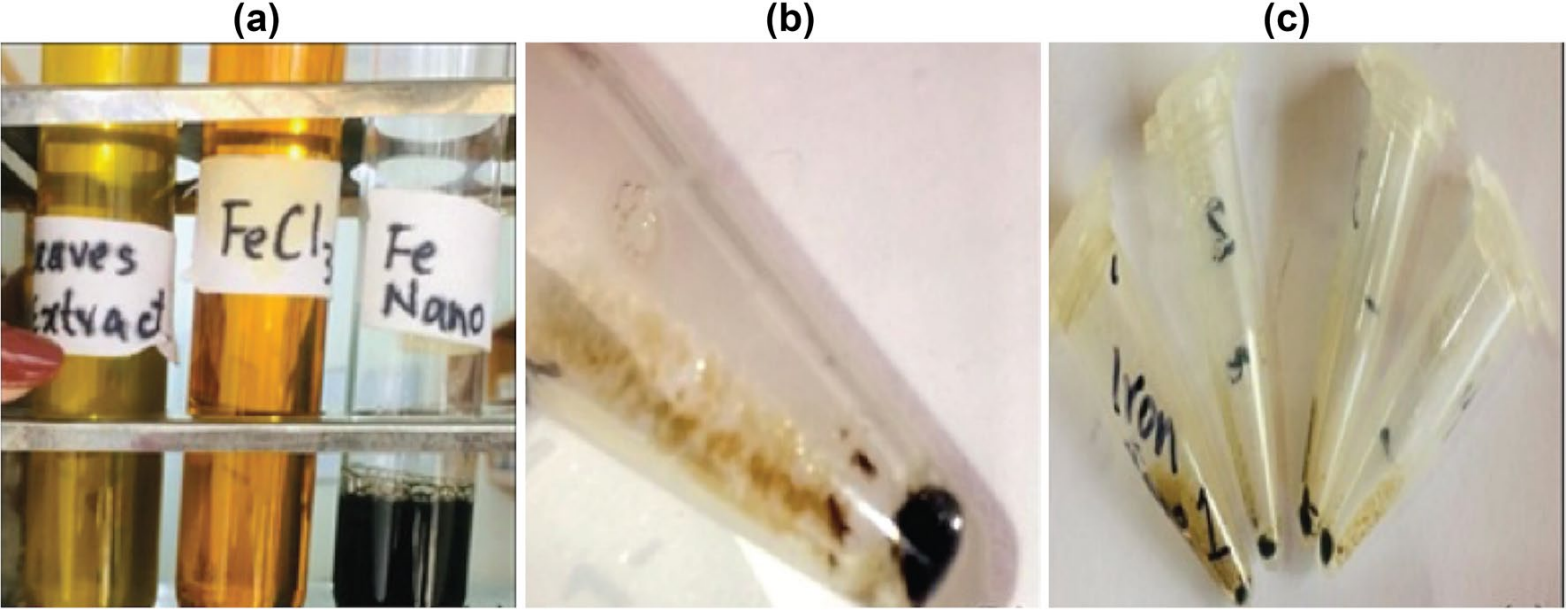
Preparation of GINPs. (**a**) Green tea leave extract, 0.1 M FeCl_3_ solution, and iron nanoparticles. (**b**, **c**) synthesized iron-nanoparticles pellet after centrifugation**.**

The Iron-nanoparticles were synthesized and characterized using the method described by Wang et al. with minor modifications^27^. For the preparation of GINPs, dried leaves (2 g) of green tea plant (*C. sinensis*) were mixed/soaked in 100 mL of de-ionized autoclaved water. The mixture was heated at 80 °C in the water bath for 20 min, and filtered via Whatman filter paper No.1. Later, 0.1 M aqueous solution of Iron Chloride (FeCl_3_) in 100 mL of de-ionized autoclaved water was prepared. After that, the filtrate of green tea solution and FeCl_3_ solution was mixed together with equal proportion by volume. Finally, to obtain Iron-nanoparticles, the mixture was centrifuged at 15,000 rpm for 15 min. For application, the pellet was washed with de-ionized autoclaved water (Fig. 1a–c).

For the initial identification, the obtained nanoparticles were subjected to UV–Vis spectroscopy (Ultraviolet–Visible Spectroscopy)^28^. For confirmation, nanoparticles were analyzed using FTIR (Fourier Transform Infrared Spectroscopy), SEM (Scanning Electron Microscope), and TEM (Transmission Electron Microscope).

### Cytotoxicity assay of iron nanoparticles

MTT Assay was performed for cytotoxicity analysis of iron nanoparticles. For this purpose, J774 cells were grown in 96-well plates. Tween 80-coated iron nanoparticles were added to the cells at defined concentrations (25, 100, 200, 300, 400, and 500 μg/mL) and incubated for three and six hours. After incubation, the media was discarded and 90 μL fresh media was added per well to the cells after thorough washing with sterile phosphate-buffered saline. 10 μL (5 mg/mL stock) of MTT reagent (3-(4,5-dimethylthiazol-2-yl)-2,5-diphenyltetrazolium bromide) was then added in wells and the plate was incubated for six hours in an incubator. After incubation, the media was discarded from the wells and dimethyl sulfoxide 100 μL was added to solubilize the formazan crystals formed. Readings were then taken in an enzyme-linked immunosorbent assay reader at 490 nm, with subtraction for plate absorbance at 650 nm^29^. Percentage viability of the cells was calculated as the ratio of mean absorbance of triplicate readings concerning mean absorbance of control wells:

$${\text{Cell}}\,{\text{viability }} = \, \left( {{\text{I}}_{{{\text{sample}}}} /{\text{I}}_{{{\text{control}}}} } \right) \, \times { 1}00.$$

### Plasmid selection

In the presented study, the pBIN.35 s-mgfp5-ER (Figure S1) plasmid was used^30^ carrying a modified GFP gene. GFP is a reporter gene of 717 bp and can be visualized at UV (395 nm) or blue light of wavelength 437 nm. Its promoter-reporter name is CaMV: GFP and its selection is kanamycin (SnapGene).

### Plasmid-iron nanoparticle complex formation

**Figure 2:**
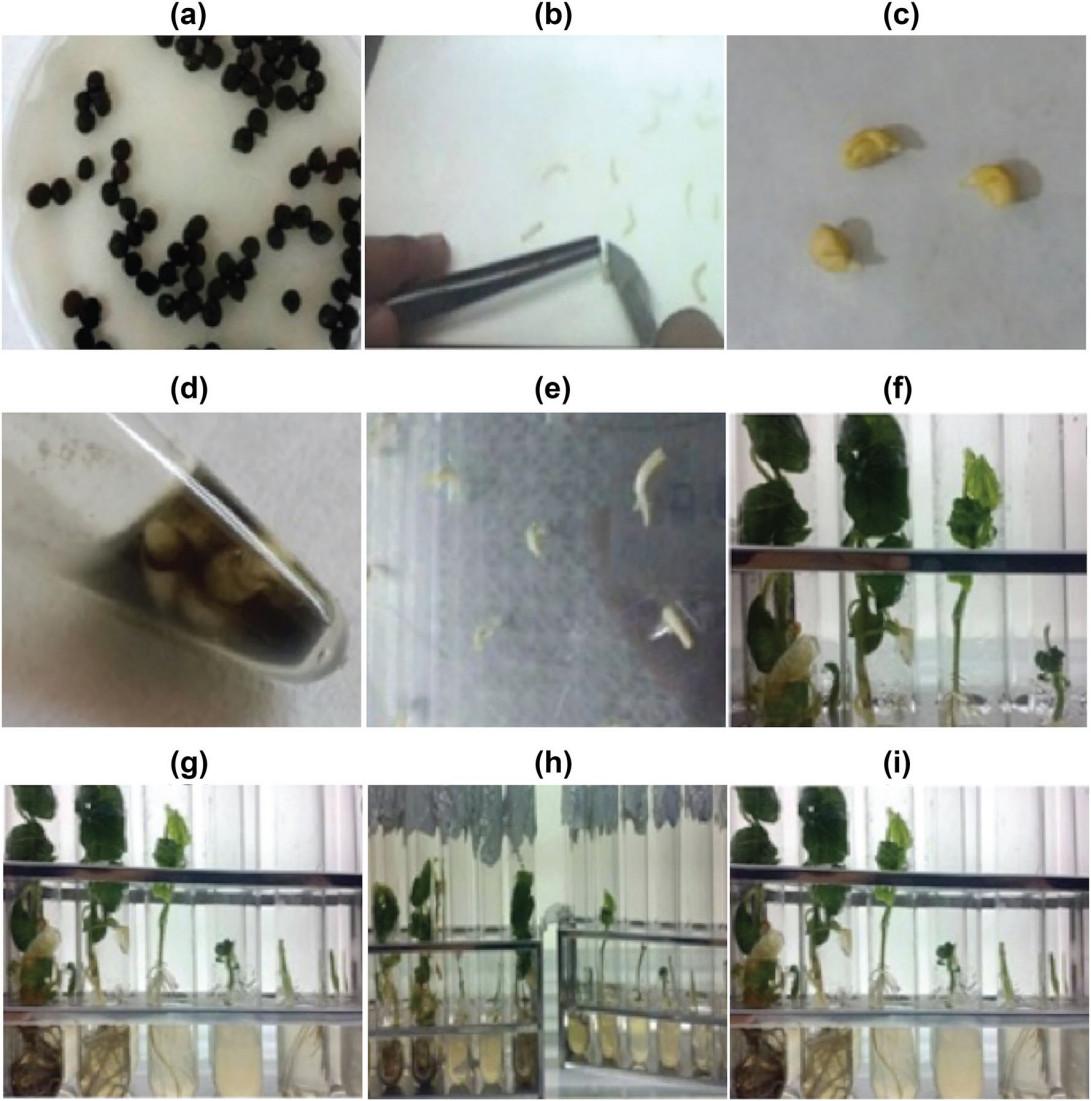
Schematic view of transformation steps. (**a**) Okra seed germination; (**b**) Isolation of Embryo; (**c**) Isolated embryo; (**d**) Plasmid-Iron Nanoparticle Complex formation; (**e**) Incubation of embryos in MS medium; (**f**) Embryo in shooting media; (**g**) plants in rooting media; (**h**) plants After 15 days; (**i**) and Different stages of plant.

Okra seeds were soaked overnight in water and then were subjected to surface sterilization with 5% sodium hypochlorite for 20 min. To obtain the embryo, the peel of seed was removed (Fig. 2a–c).

**Figure 3:**
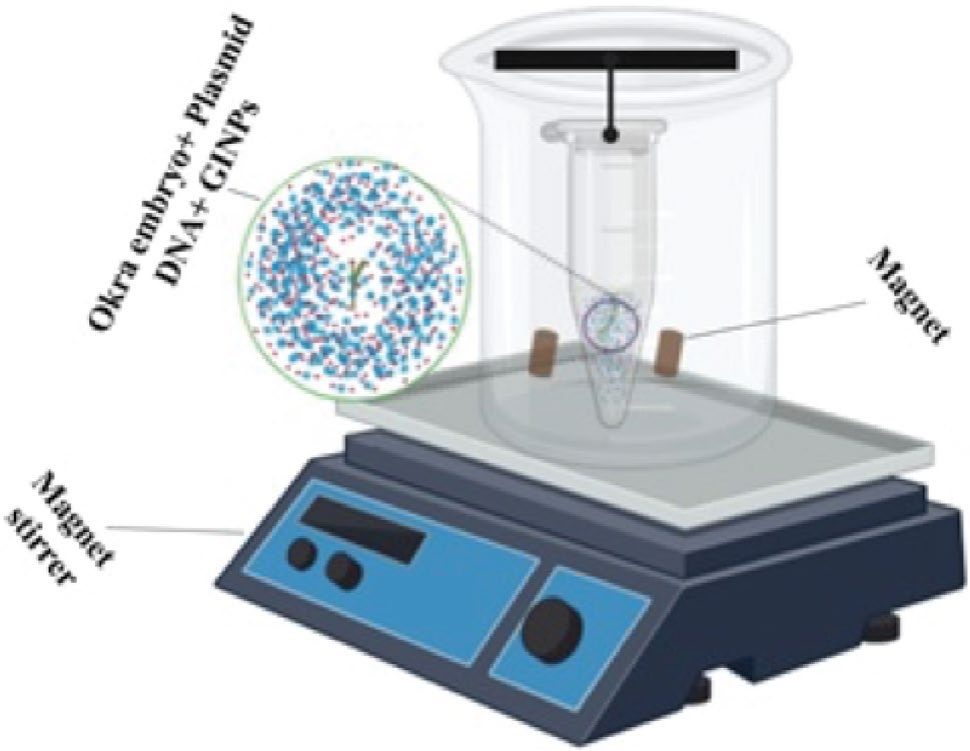
Magnetofection: illustration of DNA delivery using GINPs.

For the formation of a plasmid (pBIN.35 s-mgfp-ER)-iron nanoparticles complex, plasmid, and nanoparticles were taken in eppendorf, mixed, and kept at room temperature for 10 min. The isolated embryos dipped into a 1.5 mL tube having a plasmid-iron nanoparticle complex (Fig. 2d). For providing a magnetic field, the eppendorf was hanged in a beaker containing magnetic beads (Thermo Scientific™ Nalgene™-2.125 inches magnetic bead, catalog number: DS6630-0250). The beaker was placed in the magnetic stirrer at certain rpm and time (Fig. 3).

### Experimental design

Keeping in mind the three factors: DNA-Nanoparticle ratio (DNR), Magnetic field time (used for magnetofection), and revolution per minute (rpm), 27 treatments with 10 replicates were designed. Each treatment was subjected to a different combination of DNR, rpm, and magnetic field time (Table 1).

**Table 1:** Factors used for all 27 treatments.

No. of treatments	DNA-nanoparticle ratio (DNR)	Time (s)	Revolution per minute (RPM)
1	1:15	4	500
2	1:15	4	800
3	1:15	4	1000
4	1:15	5	500
5	1:15	5	800
6	1:15	5	1000
7	1:15	6	500
8	1:15	6	800
9	1:15	6	1000
10	1:20	4	500
11	1:20	4	800
12	1:20	4	1000
13	1:20	5	500
14	1:20	5	800
15	1:20	5	1000
16	1:20	6	500
17	1:20	6	800
18	1:20	6	1000
19	1:25	4	500
20	1:25	4	800
21	1:25	4	1000
22	1:25	5	500
23	1:25	5	800
24	1:25	5	1000
25	1:25	6	500
26	1:25	6	800
27	1:25	6	1000

The eppendorf (containing GINPs and Okra embryo) was hanged in a beaker. The beaker was placed on a magnetic stirrer for providing a magnetic field and stirring at a specific rpm and time. The illustration was prepared using the online BioRender tool (https://biorender.com/).

### Embryo germination and confirmation of GFP in okra plant

**Figure 4:**
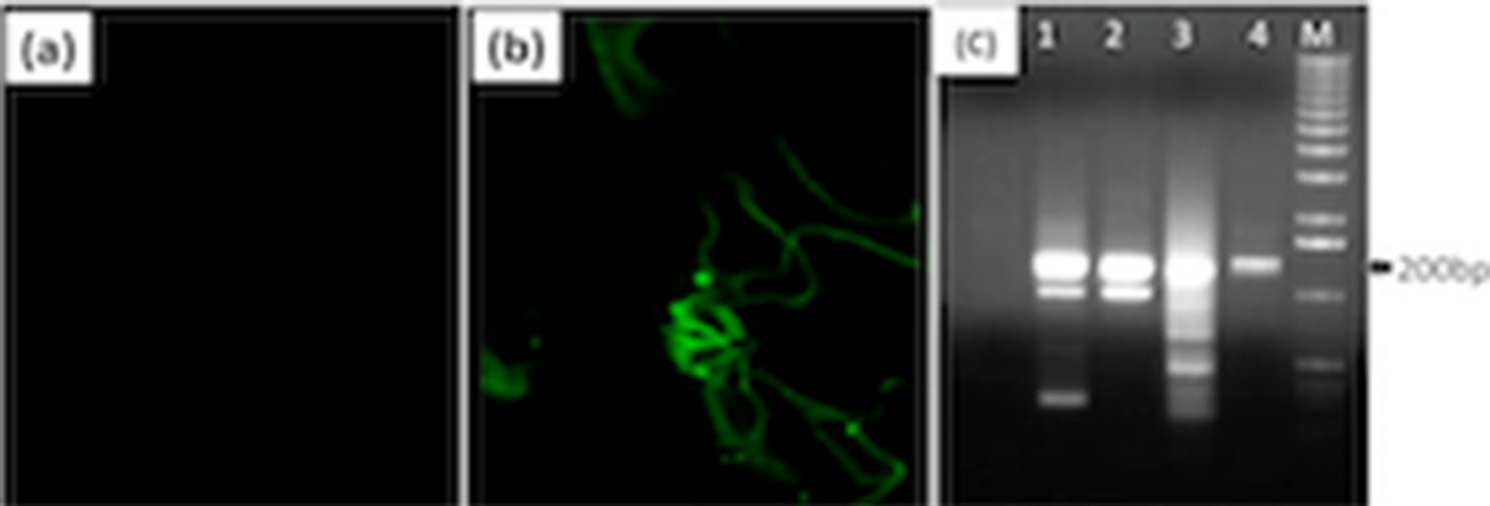
The confirmation of GFP gene transformation via Laser Scanning Confocal Microscope and PCR (**a**, **b**). The green fluorescence in transgenic Okra plants confirmed the gene delivery and its successful expression of GFP gene. (**c**) The PCR amplicons also corresponded to 200 bp as compare to the DNA ladder that confirms the delivery of GFP gene.

The MS (Murashige and Skoog) medium, containing Kanamycin, was used to grow the embryo using the tissue culture tubes. The embryo was placed in the basal media under sterilized conditions of laminar airflow (Fig. 2e). After that, the tubes were incubated at 28 °C for 21 days in a plant growth chamber (Fig. 2f–i). After germination, the presence of GFP in the okra plant was checked using a Laser Scanning Confocal Microscope (LSCM) (Fig. 4a,b) and by the amplification of the GFP (Fiqure 4c and Fig. S2) gene via polymerase chain reaction (PCR).

For the amplification of DNA, specific primers (Forward: 5′-ATGAGTAAAGGAGAAGAA-3′, Reverse: 3′-CATAAGAGAAAGTAGTG-5′) were designed to amplify the first 200 bp of the mgfp5 gene. For PCR, a reaction mixture of 25 µL was prepared to have 3 µL (20 ng/μL) template DNA, 2.5 µL 10 × Taq-polymerase buffer (Fermentas, MA, USA), 2.5 µL dNTPs (2 mM), MgCl_2_ (1.5 mM), 2 µL of forward and reverse primers, and 0.25 µl Taq-polymerase (Fermentas, MA, USA).

The thermocycler was programmed for initial degradation at 94 °C for 2 min followed by 35 cycles (94 °C for 30 s, 58 °C for 30 min, and 72 °C for 45 s). The final extension was done for 10 min at 72 °C. For confirmation, the PCR amplicons were run on 1% agarose gel (ethidium bromide; 0.5 μg/mL) and the DNA bands were observed using the digital Gel documentation system (Fig. 4c).

### Statistical analysis

**Figure 5:**
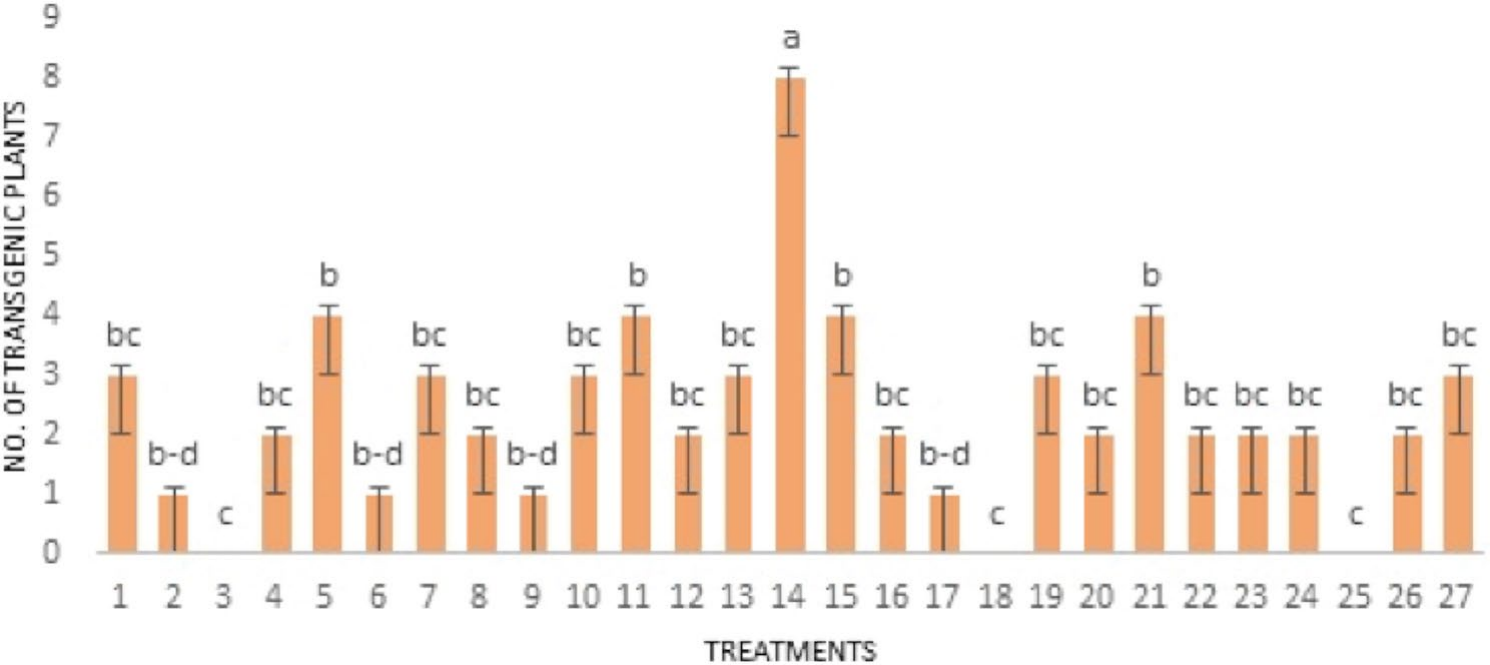
The effect of different treatments on gene delivery. Three factors: DNA to nanoparticles ratio (DNR), time of magnetofection (h), and revolution per minute (rpm). The treatment No. 14 yielded the highest number of transgenic plants that include 1:20 ration DNR, 5 h of magnetofection with 800 rpm.

The data of 270 embryos were subjected to analysis of variance (ANOVA) (Fig. 5) followed by Fisher’s protected least significant (LSD) difference test at the 5% probability level by using software Statistix 8.1 (https://www.statistix.com/). The GINPs size was measured using a TEM image with the help of ImageJ (https://imagej.net) and the frequencies of the size were calculated using OriginLab (https://www.originlab.com).

## Results

### Confirmation and characterization of GINPs

**Figure 6:**
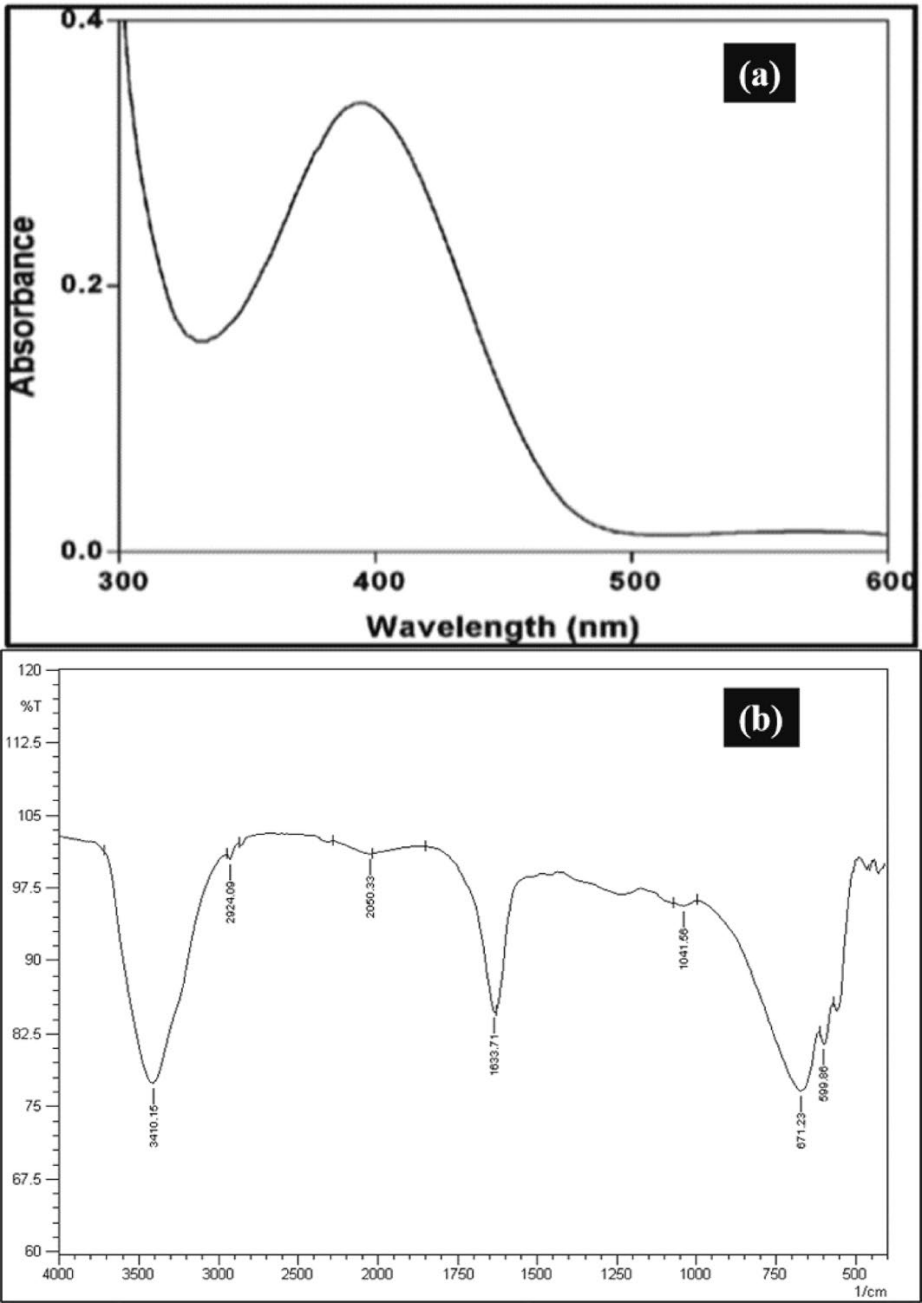
The UV–VIS (**a**) and FTIR (**b**) spectrum of the Green Iron-nanoparticles. (**a**) The absorbance of Green Iron-nanoparticles was maximum at 395 nm and that range confirms the presence of Fe–Cl complex within the sample^16^. (**b**) The FTIR spectrum of nanoparticles indicates the presence of bands at 3410, 2924, 2050, 1633, 1041, 671, and 599 cm^−1^.

Under the examination of UV–VIS Spectroscopy (Fig. 6a), the Iron-nanoparticles showed spectra between 350 and 450 nm that validate the formation of Iron-nanoparticles. The FTIR (Fourier Transformation Infrared Spectroscopy) spectrum of green Iron-nanoparticles is shown in (Fig. 6b).

**Figure 7:**
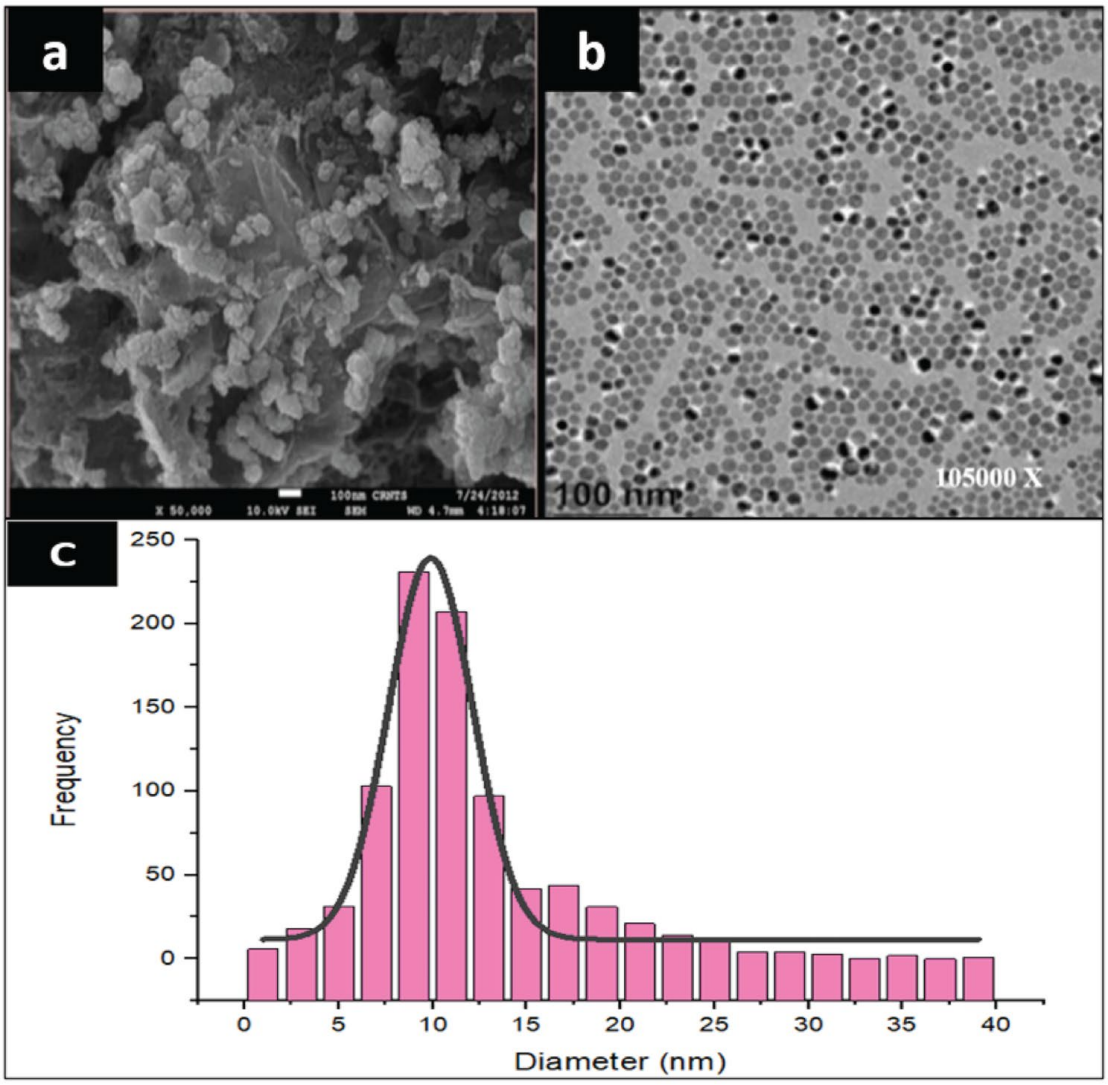
The SEM and TEM analysis of GINPs and size distribution of GINPs. (**a**) Depicts the SEM and (**b**) highlights the TEM analysis of GINPs. (**c**) The Histogram indicates that the size of GINPs was between the range of 5–40 nm. However, the highest frequencies were between 7.5 and 12.5 nm.

The FTIR spectrum of nanoparticles indicates the presence of bands at 3410, 2924, 2050, 1633, 1041, 671, and 599 cm^−1^. The mentioned bands may be due to N–H stretch, –CH, N=C, C=C, and C–Cl bonds. The presence of C–Cl bonds in the fingerprint region of the spectrum (1041, 671, and 599 cm^−1^) confirms the bonding of FeCl_3_ with the extract of green tea. The highest frequencies of GINPs size were between 7.5 and 12.5 nm as shown in Fig. 7c. The micrograph of SEM and TEM are shown in (Fig. 7a,b).

### Cytotoxicity assay of GINPs

**Figure 8:**
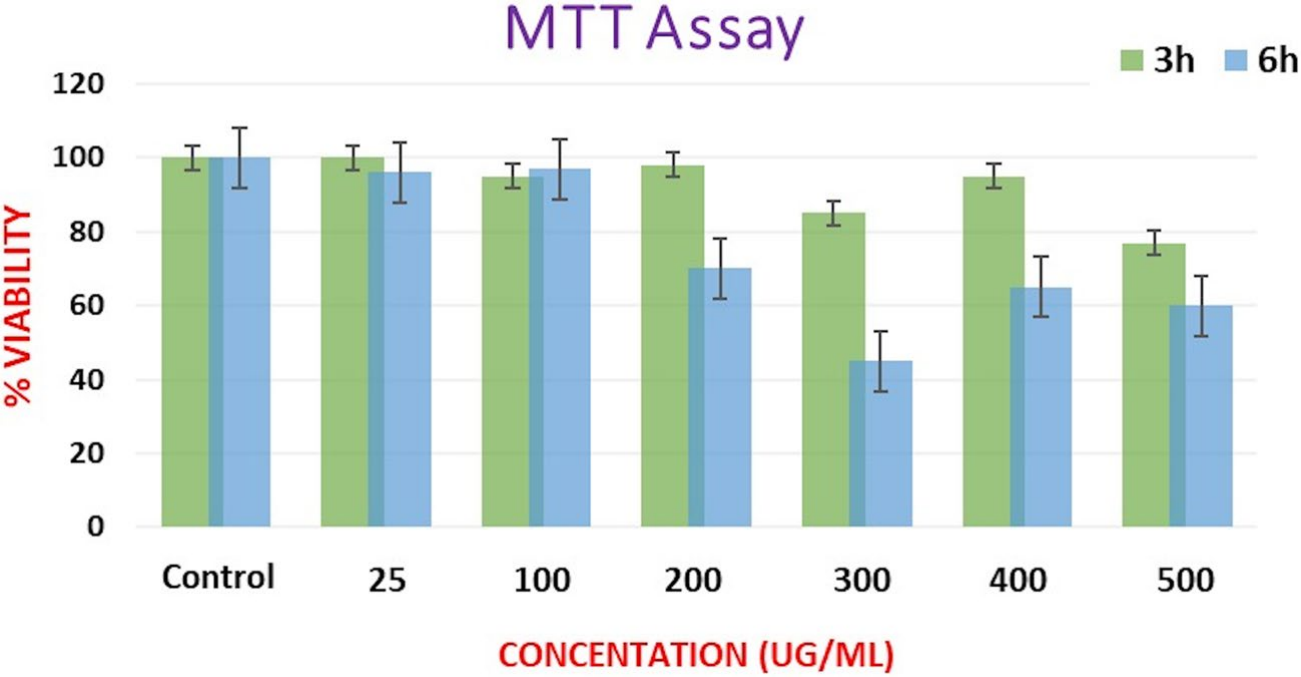
The cytotoxicity analysis of GINPs. The effects of superparamagnetic iron oxide nanoparticles on cell proliferation and viability of J774 cells as determined by MTT assay. Concentration-dependent cytotoxic effects of nanoparticles evaluated after three and six hours of incubation. Results are represented as means ± standard error of the mean. *Significant difference from control (P < 0.05).

The results of the MTT assay demonstrated that cells exposed to GINPs of mean size 30 nm for three and six hours resulted in time-dependent as well as concentration-dependent cytotoxicity. At 25 μg/mL concentration, the viability of cells at three and six hours was 100% and 95%, respectively. With increasing concentration of GINPs (25, 100, 200, 300, 400, and 500 μg/mL), the percentage viability was decreased from 100% to approximately 75% in 3 h. When the cells were incubated with the same concentration of GINPs for 6 h at 25 and 100 μg/mL, the cell viability was similar to that at three hours. In contrast, at 200 μg/mL and higher concentrations, the viability decreased significantly, ranging from 55 to 65% (Fig. 8).

### Confirmation of GFP gene transformation

The delivery of the GFP gene in plants from treatment no. 14 was confirmed as the size of PCR amplicons corresponded to the target size (200 bp) as compared to the DNA ladder (Fig. 4c). On expression in plants, the GFP produces green fluorescence when observed under LSCM. In our study, LSCM confirmed the green fluorescence in okra plants (Fig. 4b). However, there was no green fluorescence in control plants when investigated under LSCM (Fig. 4a).

### Identification of best combination for gene transformation

The statistical analysis showed that treatment no. 14 (1 µL DNA per 20 µL Iron nanoparticle solution, given the magnetic field for 5 h at 800 rpm) produced the maximum number of transgenic plants i.e., 9 out of 10 were transgenic (Fig. 5). The high Magnetofection time and rpm caused low production of transgenic plants. Even large DNR also did not increase the gene delivery into the plants.

### Experiment replication

After observing the best transformation from treatment no. 14, the experiment was reperformed with 30 okra plants subjected to treatment no. 14 i.e., 1 µL DNA per 20 µL Iron nanoparticles solution, given the magnetic field for 5 h at 800 rpm and following the same procedure of germination as mentioned above. The results obtained showed a 90% success rate as 27 out of 30 plants were transgenic.

## Discussion

**Figure 9:**
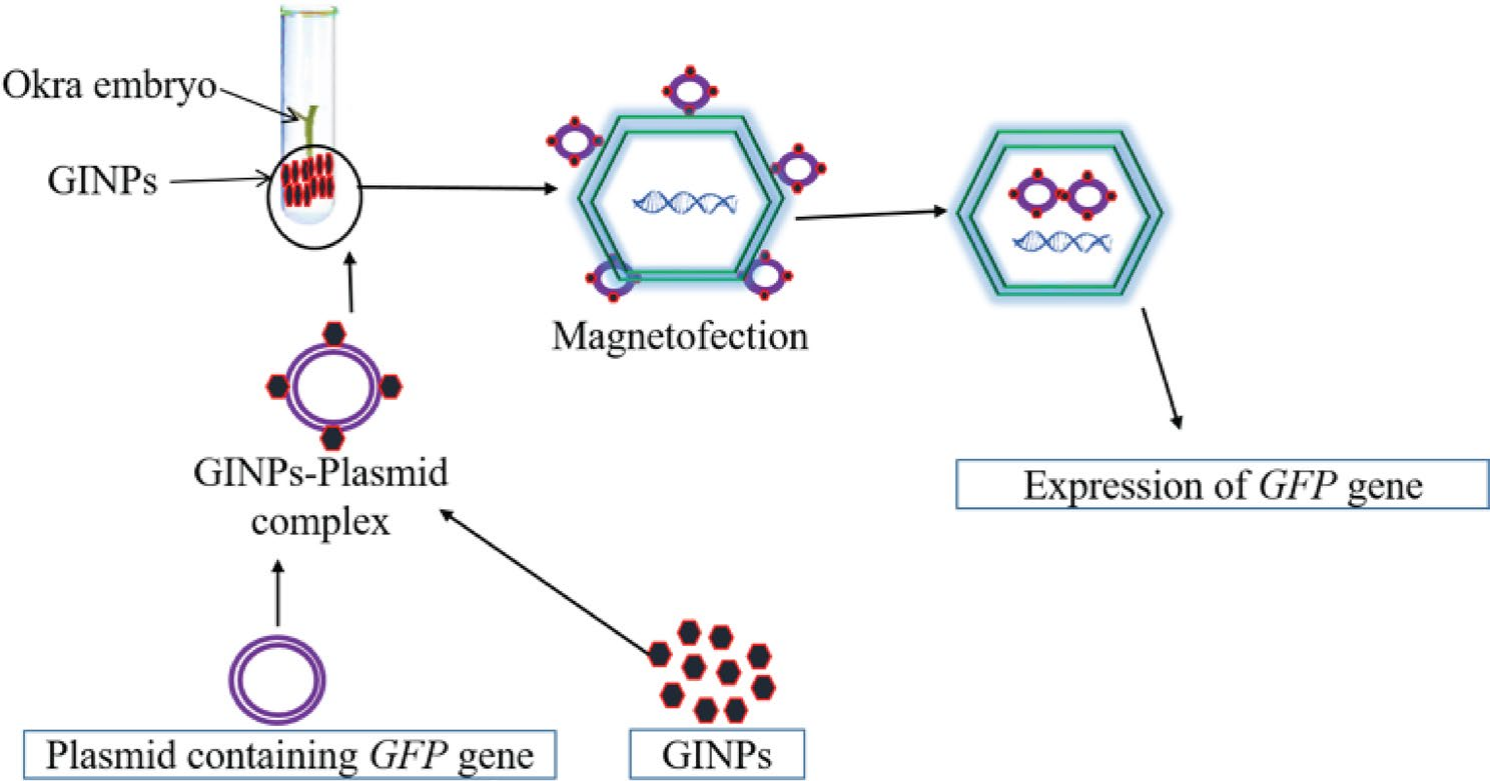
Schematic diagram showing the uptake of plasmid-nanoparticles complex by plant.

Genetic engineering of plants has paved a new direction for improved crops, accelerating the progress of the global agricultural industry. Previously, many methods have been introduced for gene transformation but each method has its own limitations^31,32^. Gene delivery, with the help of metallic nanoparticles, is a new approach in the field of biotechnology. However, these metallic nanoparticles were previously synthesized using polymers or any other coating materials^33,34^. Several reports have indicated the toxicity caused due to the use of polymers in nanoparticle-mediated drug delivery systems^35–37^. In the present study, we used the green synthesis method for the production of nanoparticles as it is eco-friendly, less toxic, more stable, and cost-effective (as instead of high energy machines and expensive chemicals, plants are used). Above all, it is time-saving as green synthesis is an one step bio-reduction process (Fig. 9)^38^.

The use of nanoparticles as molecular transporter in animals, plants, and human; nanomaterials for gene therapy and cancer therapy has been reported in previous studies. Some of the highlighted examples include; the use of Au-NP (Gold-nanoparticles) for silencing polo-loke-kinase-1(PKL1) that causes apoptosis of the damaged cell and protects the surrounding cells to get affected. In another study, to enhance the Au-NP delivery to bone marrow-derived mesenchymal cells (MSC)^39–41^. Peng et al*.* used antimicrobial peptides extracted from lactoferrin for coating Au-NP^42^. Zhi et al*.* used graphene oxide (GO) nanoparticles for delivery of microRNA21 (mir21) and Adriamycin (an anti-cancer drug) for overcoming tumor multidrug resistance, in vitro^43^. Recently, the “green approach” was used to coat the super paramagnetic iron oxide nanoparticles (SPION) with stevia plant extracts^44^. The nanoparticles genearted by green approaches are low in toxicity, biocompatible and are superior in function.

We have successfully optimized the magnetofection method for gene delivery using the GINPs. Without causing any damage to cells, Iron(III) can easily bind to the DNA and this property is useful for biotechnologists for developing a gene transformation method^45^. However, for this purpose, it requires proper magnetofection time and a specific number of revolutions and fixed DNR. Significantly, our results showed that 1:20 DNR, 800 rpm for 5 h is optimum for producing maximum number of transgenic plants via gene delivery. Contrary to this, higher rpm and time have reduced the number of transgenic plants. In a study, Lai and Singh^46^ concluded that Iron, in the presence of a magnetic field, can start a fenton reaction resulting in DNA damage. Hence, this could be the reason for yielding lesser number of transgenic plants when exposed to the magnetic field for more time.

The size of nanoparticles is also important for transfection and small-sized nanoparticles are more efficient for gene delivery^47,48^. Parallely, Wang et al.^49^ used an ionic gelation method for producing small-sized (100–200 nm) Chitosan nanoparticles for gene delivery. However, in our study, the average size of GINPs is 6 nm to 40 nm, from which the highest frequency was observed between 7.5 nm and 12.5 nm that can be more reliable for transfection.

Previously, there has been a lot of research regarding the use of nanoparticles for gene delivery in plants for crop improvement^50,51^. Demirer et al*.* in their study presented a nanomaterial-based delivery system that allows DNA delivery with high efficiency and nontoxicity or tissue damage and without transgene integration in plants^52^. Indeed, It can easily replace the traditional gene delivery methods for plants^53^. This method of gene delivery via GINPs not only shows promising consistent results and produce stable lines with higher viability than traditional gene delivery systems, but it also possesses the capability to overcome the restrictions of recalcitrant gene transmission of various plant species. So, our newly developed method could easily be used for improving crops safely in very short amount of time as it is non-toxic and cost-effective. This approach can also enable us to address abiotic limitations of the plants, ultimately making them more adaptable for cultivation on unfavourable environmental conditions.

## Conclusion

Nanoparticles-mediated gene delivery can play an important role in the establishment of a new and stable platform for genetic engineering. In the presented study, the magnetofection approach proved to be a promising method for the transformation using nanoparticles as a carrier. This study is based upon a proficient magnetofection approach with low cytotoxicity. The iron NPs were synthesized from *C. sinensis* using green synthesis, which is a more eco-friendly approach. This method of gene delivery via GINPs not only shows promising consistent results and produce stable lines with higher viability than traditional gene delivery systems, but it also possesses the capability to overcome the restrictions of recalcitrant gene transmission of various plant species. This technique can be used for transformation of current susceptible crop varieties into more resistant ones having high yield, coping with the challenges of pathogen attack and resulting yield losses in a more cost-effective and environment-friendly way by averting the use of toxic pesticides. Furthermore, it can also be applied to overcome the regional constraints due to abiotic limitations that prohibits the cultivation of economically important crops (Supplementary figures).

## Supplementary Information

Supplementary Figures.
