# Supplementary figures and images for: Magnetofection approach for the transformation of okra using green iron nanoparticles
Source: Sci Rep. 2022 Oct 4;12:16568. doi: 10.1038/s41598-022-20569-x (PMC9532403; doi:10.1038/s41598-022-20569-x)

**
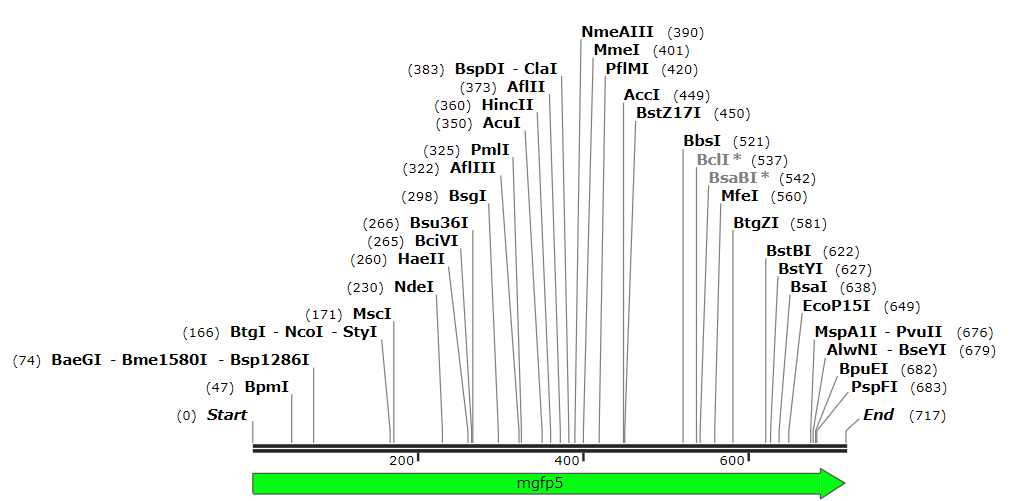
**

**Fig. S1**

DNA Map (717bp) of mgfp5

**
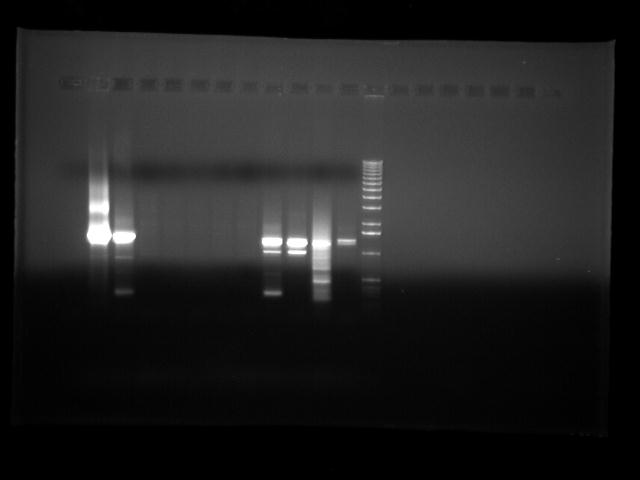
**

**Fig. S2**

Complete uncropped picture of Fig. 8c, showing PCR amplicons

Supplement: Supplementary file 1 — Supplementary Figures. [file 41598_2022_20569_MOESM1_ESM.docx]
